# Mechanistic insights into Shenqi Dihuang decoction in the treatment of immunoglobulin a nephropathy

**DOI:** 10.3389/fphar.2025.1714263

**Published:** 2026-01-20

**Authors:** Yachan Gao, Xinxin Pang, Dongdong Li, Xiaoyong Chen, Dongyang Li, Zhenyi Chen

**Affiliations:** 1 Henan Provincial Hospital of Traditional Chinese Medicine, The Second Affiliated Hospital of Henan University of Chinese Medicine, Zhengzhou, Henan, China; 2 The Second Clinical Medical College of Henan University of Chinese Medicine, Zhengzhou, Henan, China

**Keywords:** immunoglobulin a nephropathy, network pharmacology, Shenqi Dihuang decoction, single-cell sequencing analysis, target genes

## Abstract

**Background:**

Immunoglobulin A nephropathy (IgAN) is among the most prevalent glomerular disorders. Shenqi Dihuang decoction (SQD) has demonstrated therapeutic efficacy in various renal conditions, but its effects on IgAN remain insufficiently explored. The present investigation was designed to explore the potential mechanistic actions of SQD in the context of IgAN.

**Methods:**

Therapeutic targets of SQD and genes linked to IgAN were sourced from publicly available databases. An intersection analysis was performed to identify drug targets relevant to IgAN, comparing SQD target genes, differentially expressed genes (DEGs) from patients with IgAN versus healthy controls, and module genes associated with the disease. Key candidate genes were identified using feature selection techniques from machine learning, supported by experimental validation of expression patterns. Mechanistic insights were further explored through nomogram construction, gene set enrichment analysis (GSEA), immune cell infiltration profiling, molecular regulatory network reconstruction, and molecular docking simulations. Single-cell RNA sequencing identified key cell populations involved in IgAN pathogenesis, and critical gene expression patterns were assessed within these cells. Additionally, SQD’s protective effects against IgAN were validated using *in vitro* models.

**Results:**

The mechanism underlying SQD’s efficacy in IgAN may involve key molecular targets, such as *FOS*, *MCL1*, and *CCND1* (Cyclin D1 gene). These genes serve as diagnostic markers and are enriched in pathways associated with dicarboxylic acid and amino acid metabolism. Additionally, significant changes in immune cell infiltration were observed. Potential regulatory networks involving 72 miRNAs and 144 transcription factors (TFs), along with high-affinity interactions with active compounds such as cryptotanshinone, tanshinone IIA, and luteolin, were identified. Notably, proximal tubular and intercalated cells play critical roles, with FOS expression upregulated during cellular differentiation. *In vitro* experiments confirmed SQD’s significant protective effects against injury.

**Conclusion:**

Our findings proposed three potential key genes—*FOS*, *MCL1*, and *CCND1*—that may contribute to the therapeutic mechanism of SQD in IgAN, providing novel perspectives and candidates for developing targeted therapeutic approaches.

## 1 Introduction

Immunoglobulin A nephropathy (IgAN) is a glomerular disorder characterized by the deposition of IgA within the glomerular basement membrane. This pathological process is often accompanied by clinical signs such as hematuria, proteinuria, and progressive renal function decline (Rout et al., 2025). Following diagnosis, the risk of progression to end-stage renal failure increases with advancing disease stages (Rodrigues et al., 2017). Approximately 30%–45% of patients may eventually develop end-stage renal disease as their condition deteriorates (Salvadè et al., 2024). Previous studies have suggested a potential link between IgAN pathogenesis and prior upper respiratory tract infections (Wang et al., 2024a). However, the precise pathogenic mechanisms of IgAN remain incompletely understood. Evidence points to immune dysregulation and inflammatory processes as potential contributors to disease progression (Currie et al., 2022). Due to the lack of specific biomarkers, renal biopsy remains the gold standard for diagnosis, despite its invasive nature (Esteve Cols et al., 2020). Furthermore, therapeutic options are often constrained by interpatient variability and the occurrence of adverse effects (El Karoui et al., 2024; Girndt, 2024; Rajasekaran et al., 2023). A deeper understanding of the molecular mechanisms and key genetic factors involved in IgAN could pave the way for personalized, targeted therapies for affected individuals (Ding et al., 2022). Traditional Chinese Medicine (TCM), based on syndrome differentiation and personalized treatment, has been applied across a wide range of conditions, including cardiovascular diseases, dermatological reactions, and gastrointestinal disorders. Previous research (Huang et al., 2021) highlights the potential of bioactive compounds from TCM formulations for the development of targeted therapeutic strategies. TCM is widely utilized in clinical practice, with systematic investigations into its pharmacodynamics offering promising therapeutic insights (Zhang et al., 2024a). Moreover, research (Li et al., 2024) has demonstrated that bioactive components, pharmacological actions, and clinical applications of TCM play a significant role in cardiovascular disease prevention and management. Therefore, identifying potential key genes associated with IgAN through a TCM-based approach may contribute to the development of personalized, targeted treatments.

Shenqi Dihuang decoction (SQD), a TCM compound, consists of fourteen distinct herbal components: *Pseudostellaria heterophylla*, *Astragalus membranaceus*, *Paeonia lactiflora*, *Hedyotis diffusa*, *Rehmannia glutinosa*, *Dioscorea opposita*, *Salvia miltiorrhiza*, *Ligustrum lucidum*, *Eclipta prostrata*, *Cornus officinalis*, *Poria cocos*, *Alisma orientalis*, *Moutan cortex*, and *Artemisia annua*. This formulation is effective in managing various renal disorders, fever, and related symptoms, significantly reducing infection duration and slowing disease progression. Previous studies have demonstrated that SQD exerts significant therapeutic effects in the early stage of diabetic nephropathy by effectively regulating inflammatory pathways, reducing proteinuria, and protecting renal function (Wang et al., 2018; Chen et al., 1997). Meanwhile, SQD can enhance antioxidant activity and alleviate high glucose-induced injury in human renal tubular epithelial cells (HK-2) via activating the Nrf2/HO-1/GPX4 signaling axis (Wang et al., 2023). However, to date, no studies have reported on the efficacy and safety of SQD in the treatment of IgAN. Therefore, network pharmacology provides a feasible approach to explore the potential molecular targets of SQD in IgAN treatment (Dong et al., 2021; Zhang et al., 2021), which is expected to offer novel theoretical basis for the early diagnosis and targeted therapy of this disease.

Utilizing transcriptomic and single-cell RNA sequencing (scRNA-seq) datasets from public repositories, this study applied network pharmacology and bioinformatics techniques to identify key bioactive constituents and target genes of SQD. This enabled the identification of pivotal genes involved in SQD-mediated treatment of IgAN. Further analyses—including immune cell infiltration profiling, nomogram modeling, molecular network reconstruction, and molecular docking—were employed to uncover the functional roles of these genes in IgAN pathology. Additionally, scRNA-seq data from IgAN samples were analyzed to assess the expression patterns of these genes in critical cell populations. This approach provides novel insights into the mechanistic basis of SQD’s therapeutic action in IgAN and may contribute to the development of targeted treatment strategies and pharmacological agents.

## 2 Materials and methods

### 2.1 Data collection

Transcriptomic data for IgAN were sourced from the Gene Expression Omnibus (GEO) repository. The dataset GSE93798 (platform GPL22945), which includes glomerular tissue specimens from renal biopsies—20 from patients with IgAN and 22 from controls—served as the training cohort. The original author’s data preprocessing involved: the data were analyzed in R (v 3.1.3) with a custom Chip Definition File (CDF) (v 19) and a modified Affymetrix_1.44.1 package from BrainArray, followed by normalization using the Robust Multi-array Average (RMA) method and batch correction using the ComBat algorithm. Gene annotation was based on the Human Entrez Gene custom CDF (v 19). For validation, the dataset GSE37460 (platform GPL14663) was used, containing 27 IgAN samples and 9 control samples, all derived from glomerular tissues obtained through renal biopsy. The original authors’ data preprocessing was performed using the GenePattern pipeline. First, each hybridization was normalized separately with the RMA method and a Human Entrez Gene custom CDF (v 10). Subsequently, batch correction was applied using the ComBat algorithm. The final processed expression values were contained in the “VALUE” column of the output file. Additionally, scRNA-seq data with accession number GSE171314 (platform GPL20795) incorporated renal tissue from four individuals with IgAN and one healthy control.

Active compounds and molecular targets of SQD were identified. The formulation primarily includes the following herbal constituents: *Pseudostellaria radix* (Taizishen) (Huang et al., 2023), *Astragalus membranaceus* (Huangqi) (Liu et al., 2024), *Cynanchum otophyllum* (Baishao) (Yang et al., 2024), *Scleromitrion diffusum* (Baihuasheshecao) (Qi et al., 2021), *Rehmannia glutinosa* (Shengdihuang) (Jia et al., 2023), *Dioscorea polystachya* (Shanyao) (Chen et al., 2023), *Salvia miltiorrhiza* (Danshen) (Yin et al., 2021), *Ligustrum lucidum* (Nvzhenzi) (Chen et al., 2024), *Eclipta prostrata* (Hanliancao) (Tian et al., 2023), *Cornus officinalis* (Shanzhuyu) (Wang et al., 2024b), *Wolfiporia cocos* (Fuling) (Nie et al., 2020), *Alisma orientale* (Zexie) (Wu et al., 2023a), *Paeonia suffruticosa* (Mudanpi) (Liu et al., 2023a), and *Artemisia annua* (Qinghao) (Zhang et al., 2021b). The main active ingredients and targets of SQD were screened using the TCMSP database (OB ≥ 30%, DL ≥ 0.18). Target genes were retrieved from the UniProt database, and for Rehmannia glutinosa, Eclipta prostrata, Alisma orientale, and Paeonia suffruticosa, targets were sourced from the BATMAN-TCM database (score cutoff = 20, adjusted *P*-value cutoff = 0.05). After removing redundant prediction results, the network of Chinese medicines, active ingredients, and target genes was constructed using Cytoscape software (v 3.10.2) (Shannon et al., 2003).

### 2.2 Identification of IgAN-related module genes

To identify gene modules associated with IgAN, weighted gene co-expression network analysis (WGCNA) was performed using the “WGCNA” package (Langfelder and Horvath, 2008) (v 1.71). Initially, sample clustering was conducted using the “hclust” function to detect and remove outliers. A soft thresholding power was selected with the “pickSoftThreshold” function to ensure the resulting gene network conformed to a scale-free topology, with a fit index *R* ^2^ greater than 0.85. A weighted co-expression network was then generated. Genes were assigned to distinct modules using hybrid dynamic tree cutting, with each module containing a minimum of 100 genes. Highly similar modules were merged using the “mergeCloseModules” function with a median dissimilarity threshold (MEDissThres) set to 0.4. Correlations between individual modules and IgAN status were assessed using the Pearson method. The module with the highest positive correlation was selected for further analysis, applying thresholds of |correlation (cor)| > 0.3 and *P* < 0.05. Further refinement of module genes was performed by requiring |module membership (MM)| > 0.4 and |gene significance (GS)| > 0.4.

### 2.3 Identification of differentially expressed genes (DEGs)

DEGs between IgAN and control samples in dataset GSE93798 were identified using the “limma” package (Ritchie et al., 2015) (v 3.58.1), with criteria set at *P* < 0.05 and |log_2_Fold Change (FC)| > 0.5. The ten most upregulated and twenty most downregulated genes, ranked by log_2_FC values, were reported. A volcano plot generated using the “ggplot2” package (Gustavsson et al., 2022) (v 3.4.4) illustrates these DEGs, highlighting the top ten upregulated and downregulated genes. Additionally, a heatmap created with the “ComplexHeatmap” package (Gu et al., 2016) (v 2.18.0) visualizes the expression patterns of the top twenty upregulated and downregulated genes in IgAN compared to controls.

Potential IgAN-related target genes were sourced from multiple databases, including Online Mendelian Inheritance in Man (OMIM), PharmGKB, Therapeutic Target Database (TTD), and GeneCards. Records retrieved from these four repositories were merged, with duplicates removed. IgAN-associated DEGs were derived by intersecting DEGs, module genes, and known IgAN target genes. Further identification of potential therapeutic targets for IgAN was achieved by intersecting these IgAN-related DEGs with SQD target genes. The intersections were visualized using the “ggvenn” package (Mao et al., 2022) (v 0.1.10).

### 2.4 Machine learning

Candidate key genes were identified using both the LASSO and SVM-RFE algorithms. In the GSE93798 dataset, genes associated with IgAN and drug response were incorporated into the LASSO model (10-fold cross-validation) *via* the “glmnet” package (v 4.1.8), with the family set to binomial and type. measure set to class. Simultaneously, the same set of IgAN-related drug target genes was analyzed using the SVM-RFE algorithm from the “e1071” package (v 1.7.14) (Yang et al., 2022a). The algorithm employed a 10-fold cross-validation procedure. In each iteration, nine folds were used for training, and the remaining single fold was reserved for validation. Model performance was assessed by averaging the results from all 10 validation folds. The gene set corresponding to the iteration with the lowest average error rate was ultimately selected as the final set of feature genes. The resulting candidate genes from both computational approaches were intersected to obtain a refined gene set, visualized using the “ggvenn” package (v 0.1.10).

### 2.5 Protein-protein interaction (PPI) network construction

To examine protein-level interactions among IgAN-related drug targets, a PPI network was constructed using the STRING database, with an interaction confidence score threshold set to 0.4. The network was visualized and analyzed with Cytoscape software (v 3.10.2).

### 2.6 Function and pathways of IgAN-associated drug targets

Functional and pathway enrichment analyses for IgAN-associated drug targets were conducted *via* Gene Ontology (GO) and Kyoto Encyclopedia of Genes and Genomes (KEGG) assessments using the “clusterProfiler” package (v 4.10.0) (Wu et al., 2021) and the “org.Hs.e.g.,.db” annotation package (v 3.18.0), with a significance threshold of *P* < 0.05. The GO analysis covered cellular components (CC), biological processes (BP), and molecular functions (MF), with results visualized *via* the “GOplot” package (v 1.0.2) (D et al., 2022). The top five most significantly enriched terms from each GO category and the top ten KEGG pathways, ranked by ascending *P*-value, were presented. Furthermore, a network illustrating interactions between active ingredients and their corresponding IgAN-related drug targets was constructed and visualized in Cytoscape (v 3.10.2).

### 2.7 Identification of potential key genes and receiver operating characteristic (ROC) analysis

Potential key genes were identified based on differential expression and consistent directional changes between IgAN and control samples. For the GSE93798 and GSE37460 datasets, expression differences were evaluated using the Wilcoxon test (*P* < 0.05), with results visualized using the “ggplot2” package (v 3.4.4). Genes exhibiting significant differential expression and consistent regulation patterns across both datasets were selected as key candidates. The diagnostic performance of these potential key genes was further assessed through ROC analysis across all samples in GSE93798 and GSE37460. ROC curves were generated using the “pROC” package (v 1.18.5) (Robin et al., 2011), with an area under the curve (AUC) greater than 0.7 indicating strong discriminative ability.

### 2.8 Construction of nomogram

A nomogram prediction model was developed to assess the diagnostic potential of the identified potential key genes. Using the “rms” package (v 6.7–1) (Xu et al., 2023), the model was constructed with data from all samples in the GSE93798 dataset. Each potential key gene was assigned a specific point value within the nomogram, and the total score was calculated by summing the individual points, reflecting the probability of IgAN occurrence. Higher scores corresponded to an increased risk of the disease. The model’s predictive accuracy and clinical utility were evaluated using a calibration curve generated through the “calibrate” function, and decision curve analysis (DCA) was performed with the “rmda” package (v 1.6) (Kerr et al., 2016).

### 2.9 Enrichment analysis of potential key genes

Gene Set Enrichment Analysis (GSEA) was performed to identify biological pathways associated with the potential key genes, using the reference sets “c2. cp.kegg.v7.4. entrez.gmt” and “c5. all.v7.4. entrez.gmt.” Correlation analyses between potential key genes and all other genes in the GSE93798 dataset were performed for each sample, and the results were visualized using the “corrplot” package (v 0.92) (Liu et al., 2022). Subsequently, GSEA was conducted using the “clusterProfiler” package (v 4.10.0), with thresholds set at |NES| > 1 and adjusted *P*-value <0.05. The top five most significant enrichment outcomes are reported.

### 2.10 Immune infiltration analysis

Immune cell infiltration differences between patients with IgAN and controls were assessed using the CIBERSORT algorithm (Li et al., 2023) to estimate the relative abundance of 22 immune cell types across all samples in the GSE93798 dataset. The analysis was performed with the “IOBR” package (v 0.99.8) (Zeng et al., 2021), and results were visualized using “ggplot2” (v 3.4.4), applying a significance threshold of *P* < 0.05. Differences in immune cell composition between IgAN and control groups were evaluated using the Wilcoxon test (*P* < 0.05). Additionally, Spearman correlation analysis, conducted with the “psych” package (v 2.4.3) (Robles-Jimenez et al., 2021), was used to assess correlations between differentially abundant immune cells and potential key genes, with thresholds set at |correlation coefficient| > 0.3 and *P* < 0.05.

### 2.11 Prediction of regulatory molecular

To explore the upstream regulatory mechanisms of the potential key genes, a molecular interaction network was constructed. Potential miRNAs targeting these potential key genes were predicted using the miRDB and MirDIP databases. High-confidence miRNA candidates were identified by overlapping predictions from both databases, and the miRNA–mRNA regulatory network was visualized using Cytoscape (v 3.10.2). Transcription factors (TFs) that may regulate the potential key genes were predicted using the NetworkAnalyst database.

### 2.12 Molecular docking

Active compounds corresponding to potential key genes were selected for molecular docking based on their oral bioavailability (OB) ranking to assess their binding affinity with target proteins. The three-dimensional structures of the proteins encoded by potential key genes were retrieved from the Protein Data Bank (PDB). Prior to docking, small molecules and water were removed from the protein structures. Hydrogen atoms were added, and partial charges were assigned using AutoDock Tools to prepare the proteins for docking simulations. The two-dimensional structural formulas of the target compounds were obtained from the PubChem database. AutoDock Tools was used to verify charge distribution and identify rotatable bonds within each ligand. Docking grid parameters were defined based on the active site coordinates of the receptor proteins. Molecular docking was performed using AutoDock Vina, and the conformation with the lowest binding free energy was visualized and exported using PyMol (v 2.6.0a0) (Kagami et al., 2020). To validate the specificity of the molecular docking between FOS and Tanshinone IIA, N-nitrosodiethylamine was used as a negative control in the docking analysis. The three-dimensional structural data for N-NITROSODIETHYLAMINE were obtained from the PubChem database. Subsequently, the protein and ligand were submitted together to the CB-Dock2 online server (https://cadd.labshare.cn/cb-dock2/index.php) for docking simulation and binding free energy calculation.

### 2.13 The scRNA-seq analysis

All scRNA-seq data were processed using the “Seurat” package (v 5.0.1) (Hao et al., 2021). The analysis pipeline consisted of the following steps: First, gene expression levels were normalized across all cells using the LogNormalize method with a scale factor of 10,000 via the NormalizeData function. Then, the expression matrix was centered and scaled using the ScaleData function to mitigate the influence of extreme expression variances. It is noteworthy that data integration was not performed on this dataset; therefore, no batch effect correction was applied. Finally, low-quality cells were rigorously filtered out through strict quality control measures. For the GSE171314 dataset, cells were retained if they met the following criteria: total RNA counts above 20,000, unique gene features (nFeature RNA) between 200 and 7,000, and a mitochondrial gene percentage below 30%, calculated using the “PercentageFeatureSet” function.

After quality control, the scRNA-seq dataset underwent normalization and feature selection with the “NormalizeData” and “FindVariableFeatures” functions to identify genes with high intercellular variability. The top 2,000 most variable genes were selected for further analysis, with the ten genes exhibiting the greatest variation being highlighted.

Principal component analysis (PCA) was performed for dimensionality reduction while retaining the majority of the dataset’s variance. The statistical significance of the top 50 principal components (PCs) was evaluated using the “JackStraw” function, with components having *P*-values below 0.05 retained. An elbow plot was generated using the “ElbowPlot” function to determine the optimal number of components for subsequent analysis.

The t-distributed stochastic neighbor embedding (t-SNE) algorithm was then applied for nonlinear dimensionality reduction of the selected PCs. Cell clustering was conducted using the “FindClusters” function, with a resolution parameter set to 0.5. DEGs for each cluster were identified using the “FindAllMarkers” function, applying thresholds of |log_2_FC| > 0.5 and an adjusted *P*-value <0.05 to annotate cell types within IgAN and control renal biopsy specimens.

Cell type annotation was performed using the “SingleR” package (v 2.0.0) (Zhang et al., 2022), with references to the CellMaker database, HumanPrimaryCellAtlasData, BlueprintEncodeData, ImmuneCellExpressionData, and established marker genes from relevant literature (Tang et al., 2021; Lake et al., 2023). A bubble plot was generated to visualize marker gene expression patterns across distinct cell clusters. Following annotation, cells were grouped and displayed according to their sample origin, and the relative abundance of each cell type was compared between IgAN and control groups.

To identify pivotal cell populations, expression levels of potential key genes were compared between IgAN and control samples across all annotated cell types, with results visualized using violin plots and bubble diagrams. Differential expression among cell types was assessed using the Wilcoxon test (*P* < 0.05). Cell clusters exhibiting statistically significant expression differences were identified as key cellular subsets.

Pseudotemporal trajectory analysis was performed to investigate gene expression dynamics during cellular differentiation within key cell populations. PCA was applied using the “RunPCA” function for clustering and dimensionality reduction of these cells. Grouping into distinct clusters was then achieved using the “FindClusters” function, with a resolution parameter of 0.4. These subgroups were subjected to pseudotime ordering analysis using the “Monocle” package (v 2.30.1) (Qiu et al., 2017) to infer developmental trajectories.

### 2.14 Construction of animal models

This study employed 20 specific pathogen-free (SPF)-grade Sprague-Dawley (SD) rats to establish an IgAN model via combined oral administration of bovine serum albumin (BSA) with subcutaneous injection of CCl_4_ and tail vein injection of lipopolysaccharide (LPS), following the method reported by Wan et al. (Wan et al., 2022). With minor modifications. The specific steps were as follows: During weeks 1–12, rats received BSA (600 mg kg^-1^) via oral gavage every other day and a weekly subcutaneous injection of 0.1 mL CCl_4_ mixed with 0.3 mL castor oil. LPS (0.25 g L^-1^) was administered via tail vein injection at 0.2 mL on weeks 6, 8, 10, and 12. At week 8, 24-h urinary red blood cells were detected; positive results were considered successful establishment of the IgAN model. Rats with successful modeling were randomly divided into treatment, control, and blank groups. Starting from week 9, daily oral intervention was administered for 4 consecutive weeks. The treatment group received oral administration of Shenqi Dihuang Decoction at a dose equivalent to 1.3 g kg^-1^·d^-1^ based on body surface area. The control group received Tripterygium glycosides suspension (mass concentration 1.875 g L^-1^, prepared with physiological saline, freshly prepared for each use). The sham group received an equal volume of saline (5 mL kg^-1^). After the final gavage, rats were placed in metabolic cages for 24-h urine collection, with samples stored at −20 °C. Under conditions of food deprivation but free water access, blood was collected via the abdominal aorta. Serum was separated by centrifugation at 3,000 rpm for 15 min and stored at −20 °C. Both kidneys were removed: the right kidney was fixed in 4% paraformaldehyde, while a portion of the left kidney was rapidly frozen in liquid nitrogen for cryosectioning. The remainder was stored at −80 °C for subsequent Reverse transcription quantitative polymerase chain reaction (RT-qPCR) analysis.

The composition of the Ginseng-Astragalus-Rehmannia Decoction is as follows: 15 g of Codonopsis pilosula, 15 g of Astragalus membranaceus, 15 g of Paeonia lactiflora, 12 g of Hedyotis diffusa, 12 g of Rehmannia glutinosa, 15 g of Dioscorea opposita, 15 g of Salvia miltiorrhiza, 12 g of Ligustrum lucidum, 15 g of Eclipta prostrata, 15 g of Cornus officinalis, 12 g of Poria cocos, Alisma orientale 12 g, Moutan bark 9 g, Artemisia annua 12 g.

### 2.15 RT-qPCR

To evaluate the protective effects of SQD against IgAN *in vitro*, RT-qPCR was performed. Five paired tissue specimens from a rat model were obtained from Henan Province Hospital of Traditional Chinese Medicine, with five samples each from IgAN-induced and SQD-treated groups. The study followed the ARRIVE 2.0 guidelines and was approved by the Ethics Committee of Henan Provincial Hospital of Traditional Chinese Medicine (approval number: PZ-HNSZYY-2024-035). Total RNA extraction from the five sample pairs was carried out using TRIzol reagent (Ambion, U.S.A.) for subsequent RT-qPCR analysis. RNA concentration was measured using a NanoPhotometer N50 spectrophotometer. Messenger RNA was then reverse-transcribed into complementary DNA (cDNA) using a commercial kit (Servicebio, Wuhan, China). RT-qPCR amplification was performed according to established protocols, including reaction conditions, master mix composition, and primer sequences (Table 1). Relative expression levels of potential key genes in IgAN versus SQD-treated samples were quantified using the 2^–ΔΔCt method, with GAPDH as the internal reference gene for normalization. All measurements were performed in triplicate technical replicates to ensure accuracy and reproducibility. Data analysis and visualization were conducted using GraphPad Prism 10. Comparisons between the two groups were performed using the t-test, with *P* < 0.05 considered statistically significant.

**TABLE 1:** Table of RT-qPCR primer sequences.

Primers	Sequences
FOS F	GGC​AGA​AGG​GGC​AAA​GTA​GA
FOS R	TTG​GCA​ATC​TCG​GTC​TGC​AA
MCL1 F	AGA​TGG​CGT​AAC​AAA​CTG​GGG
MCL1 R	CGC​CTT​CTA​GGT​CCT​GTA​CG
CCND1 F	TCA​AGT​GTG​ACC​CGG​ACT​G
CCND1 R	GTG​GCC​TTG​GGA​TCG​ATG​TT
GAPDH F	GGC​CGG​AGA​CGA​ATG​GAA​ATT​A
GAPDH R	CCA​AAT​CCG​TTC​ACA​CCG​AC

### 2.16 Statistical analysis

All statistical and bioinformatic analyses were conducted in the R programming environment (v 4.3.2). Differences between two-group comparisons were assessed using Wilcoxon rank-sum tests, with a significance threshold set at *P* < 0.05.

## 3 Results

### 3.1 Identification of IgAN-related module genes

**FIGURE 1:**
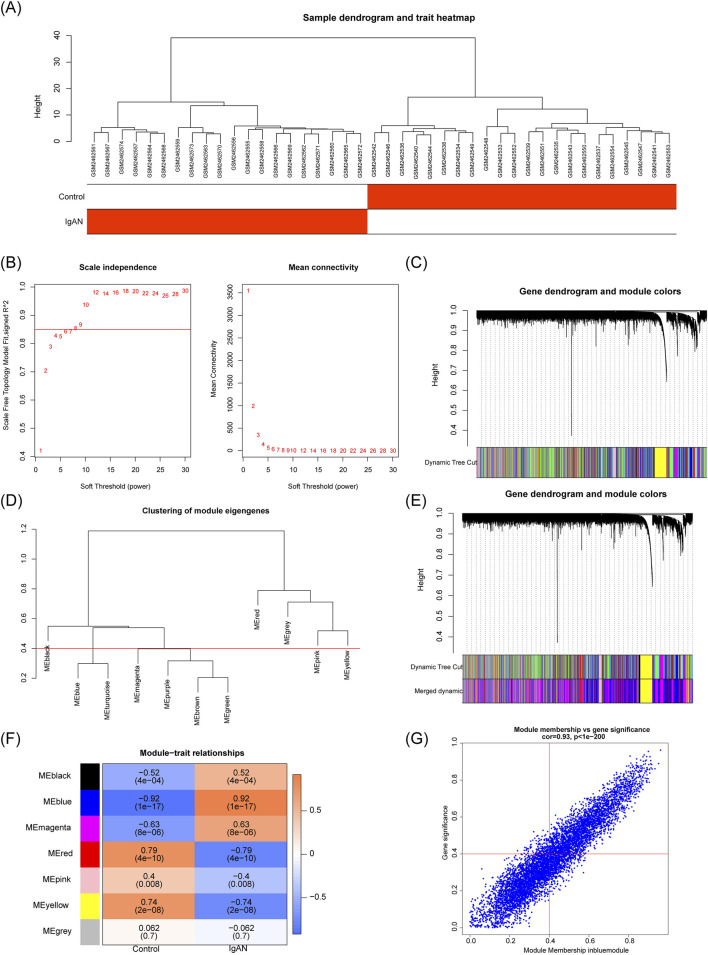
Identification of module genes associated with IgAN via WGCNA. **(A)** Clustering dendrogram of samples, indicating absence of outliers. **(B)** Analysis of scale-free topology fit indices used for determining soft-thresholding power (power = 8). **(C)** Dendrogram of genes with assigned module colors. **(D)** Clustering of module eigengenes prior to merging. **(E)** Clustering of module eigengenes following merging. **(F)** Correlation heatmap between modules and clinical traits, with MEblue exhibiting the highest association. **(G)** Scatterplot illustrating gene significance against module membership within the MEblue module.

The sample clustering results showed no outlier specimens (Figure 1A). A soft-thresholding power of 8 was chosen to achieve a scale-free topology fit (*R* ^2^ = 0.85), while maintaining mean connectivity close to zero (Figure 1B). The construction of a weighted co-expression network matrix initially produced 10 distinct modules (Figure 1C). After consolidating highly similar modules, six cohesive modules were retained for further analysis (Figures 1D,E). Among these, the MEblue module exhibited the strongest positive correlation with the trait of interest (cor = 0.92, *P* < 0.05) (Figure 1F). Applying thresholds for MM and GS identified 2,817 high-confidence key module genes (Figure 1G).

### 3.2 Identification of IgAN-related DEGs

**FIGURE 2:**
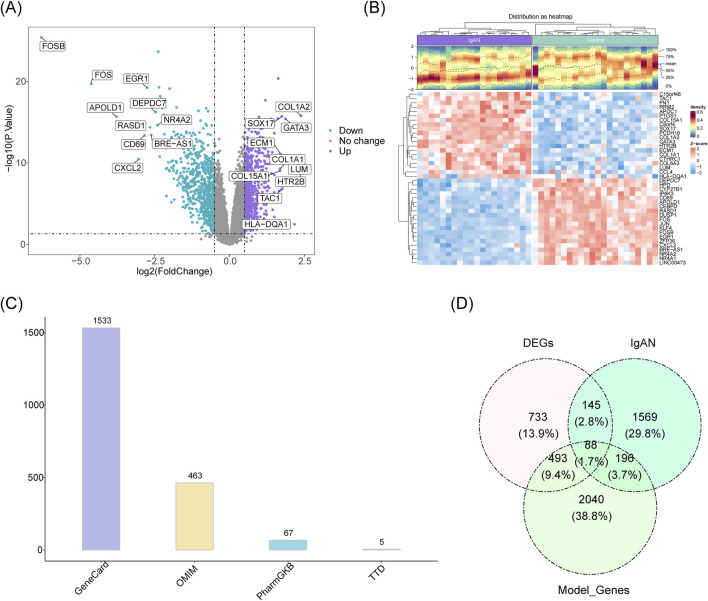
Analysis of differential expression in IgA nephropathy. **(A)** Volcano plot displaying differentially expressed genes (DEGs), annotated with the ten most significantly upregulated and downregulated genes. **(B)** Heatmap illustrating the expression patterns of the top 20 upregulated and downregulated genes. **(C)** Venn diagram representing target genes associated with IgAN identified across four distinct databases. **(D)** Overlap among DEGs, module genes from WGCNA, and IgAN-related target genes.

Differential expression analysis revealed 1,459 significantly altered genes in the IgAN group, comprising 725 upregulated and 734 downregulated genes. A heatmap depicting the ten most strongly upregulated and downregulated genes among these DEGs was generated (Figure 2A). An additional heatmap visualizes the top 20 upregulated and downregulated genes in IgAN samples compared to controls (Figure 2B). A total of 463 target genes associated with IgAN were retrieved from the OMIM database, 1,533 candidate genes were identified from GeneCards, and the TTD and PharmGKB databases contributed 5 and 67 target genes, respectively. After integration and removal of duplicates, a final set of 1,998 IgAN-related target genes was compiled (Figure 2C). Eighty-eight genes associated with IgAN were identified through the intersection of DEGs, module genes, and known IgAN-related targets (Figure 2D).

### 3.3 Identification and function of IgAN-related drug target genes

**FIGURE 3:**
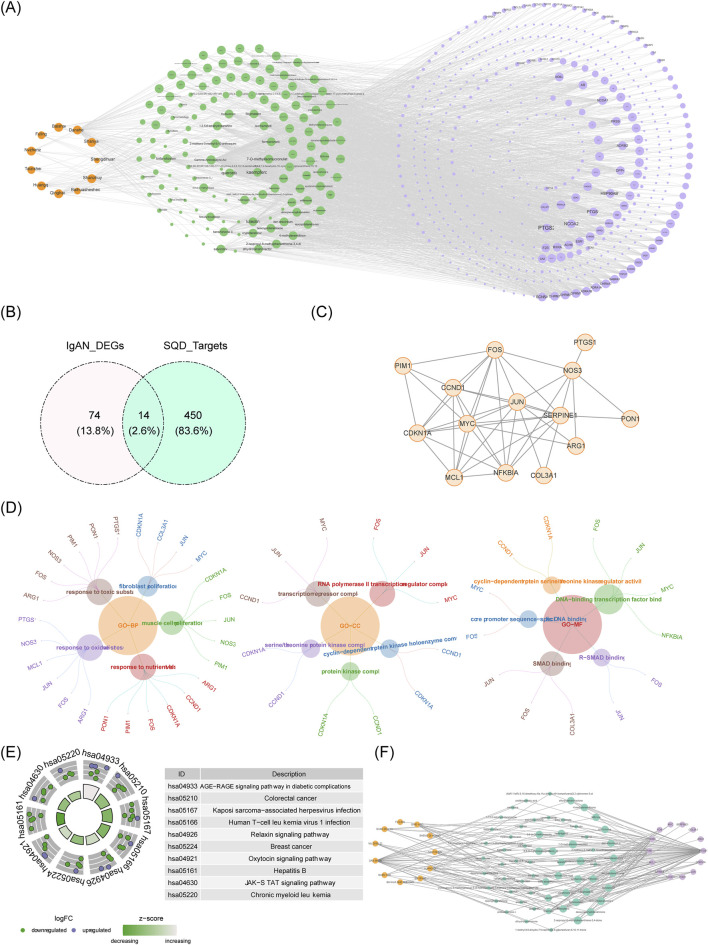
Network and functional profiling of SQD targets. **(A)** Network representation illustrating associations among herbs, bioactive constituents, and target molecules in SQD. **(B)** Venn diagram showing overlapping genes between differentially expressed genes related to IgAN and targets of SQD. **(C)** Protein-protein interaction (PPI) network constructed using drug targets associated with IgAN. **(D)** Gene Ontology (GO) enrichment analysis across biological processes, cellular components, and molecular functions. **(E)** Enrichment analysis of KEGG signaling pathways. **(F)** Integrated network connecting herbal components, active ingredients, and targets implicated in IgAN.

A total of 136 bioactive compounds and 464 corresponding target genes were incorporated into a comprehensive network depicting traditional medicine compounds, active ingredients, and SQD target genes (Figure 3A; Table 2). Intersection analysis between IgAN-related DEGs and SQD target genes revealed 14 overlapping genes (Figure 3B). PPI network analysis showed that these 14 IgAN-related drug target genes, including MYC, JUN, and SERPINE1, engage in functional interactions at the protein level (Figure 3C). GO enrichment analysis identified 702 significantly enriched pathways, comprising 625 BPs, 25 CCs, and 52 MFs (Online Resource 1). The tree map revealed that BPs were predominantly enriched in muscle cell proliferation and fibroblast proliferation, CCs were mainly associated with protein kinase complexes and transcription repressor complexes, and MFs showed significant enrichment in SMAD binding and DNA-binding transcription factor binding activities (Figure 3D). KEGG pathway analysis identified 88 significantly enriched signaling pathways, including relaxin, JAK-STAT, and oxytocin signaling pathways (Figure 3E, Online Resource 2). Finally, a comprehensive network integrating traditional medicines, active ingredients, and IgAN target genes was constructed (Figure 3F).

**TABLE 2:** Statistics on bioactive constituents of SQD and corresponding target genes.

Herbal component (Chinese name)	Number of bioactive constituents	Number of target genes	Number of ingredient-target interaction pairs
Taizishen	8	93	125
Huangqi	20	213	462
Baishao	13	92	123
Baihuasheshecao	7	187	256
Shanyao	16	78	144
Danshen	65	136	932
Nvzhenzi	13	196	352
Shanzhuyu	20	70	130
Fuling	15	25	30
Qinghao	22	214	510
Shengdihuang	4	216	217
Hanliancao	1	-	-
Zexie	-	-	-
Mudanpi	-	-	-

1. Data were retrieved from TCMSP, database (screening criteria: Oral Bioavailability [OB] ≥ 30%, Drug-Likeness [DL] ≥ 0.18) for most herbs; targets of Rehmannia glutinosa, Eclipta prostrata, Alisma orientale, and Paeonia suffruticosa were from BATMAN-TCM (score cutoff = 20, adjusted P-value cutoff = 0.05). 2. “-” indicates no qualified bioactive constituents/targets identified under the above screening criteria.

### 3.4 Identification of potential key genes

**FIGURE 4:**
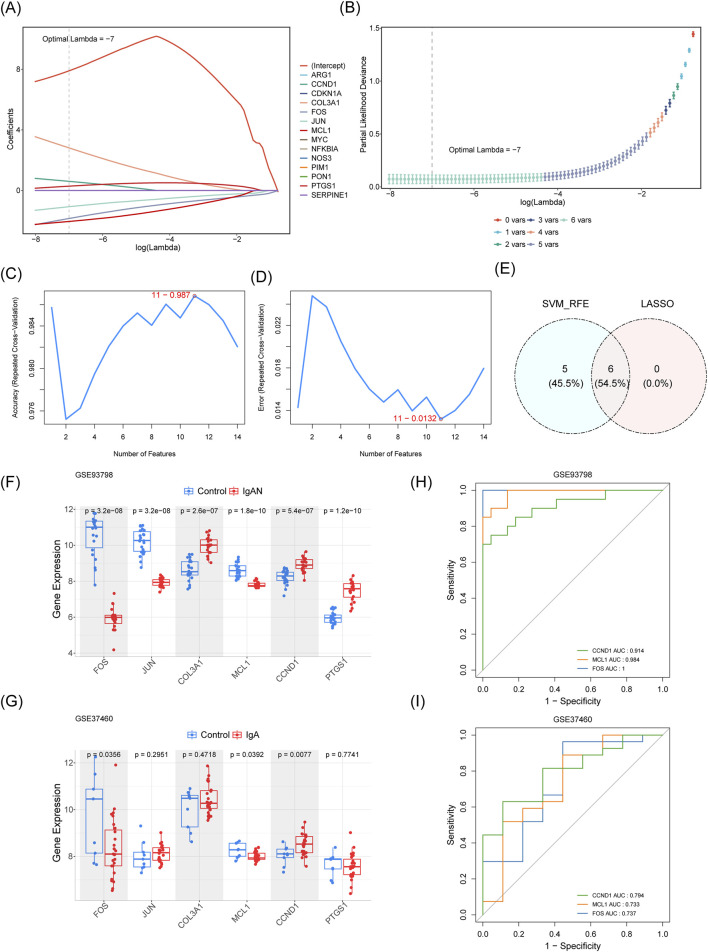
Identification of hub genes. **(A)** Coefficient profiles generated by the LASSO regression algorithm. **(B)** Ten-fold cross-validation curve for optimal lambda selection in LASSO modeling. **(C)** Feature selection ranking via Support Vector Machine-Recursive Feature Elimination (SVM-RFE). **(D)** Cross-validation results during the SVM-RFE process. **(E)** Overlap of feature subsets identified by both LASSO and SVM-RFE approaches. **(F,G)** Expression patterns of candidate hub genes across training and independent validation cohorts. **(H,I)** Receiver operating characteristic (ROC) curves evaluating diagnostic performance of potential key genes in each dataset.

Application of the LASSO algorithm with lambda. min = −7 identified a set of six genes (Figures 4A,B). The SVM-RFE method independently identified eleven candidate genes (Figures 4C,D). The intersection of results from both machine learning approaches yielded six consensus genes, which were selected for further investigation (Figure 4E). In both the GSE93798 and GSE37460 datasets, expression levels of FOS and MCL1 were significantly reduced in IgAN samples compared to controls, whereas CCND1 exhibited markedly elevated expression in the IgAN group (Figures 4F,G). Given these consistent differential expression patterns, FOS, MCL1, and CCND1 were designated as potential key genes for this study. ROC analysis demonstrated strong diagnostic performance for these potential key genes in both datasets, with AUC values surpassing 0.7 (Figures 4H,I).

### 3.5 Construction and validation of the nomogram

**FIGURE 5:**
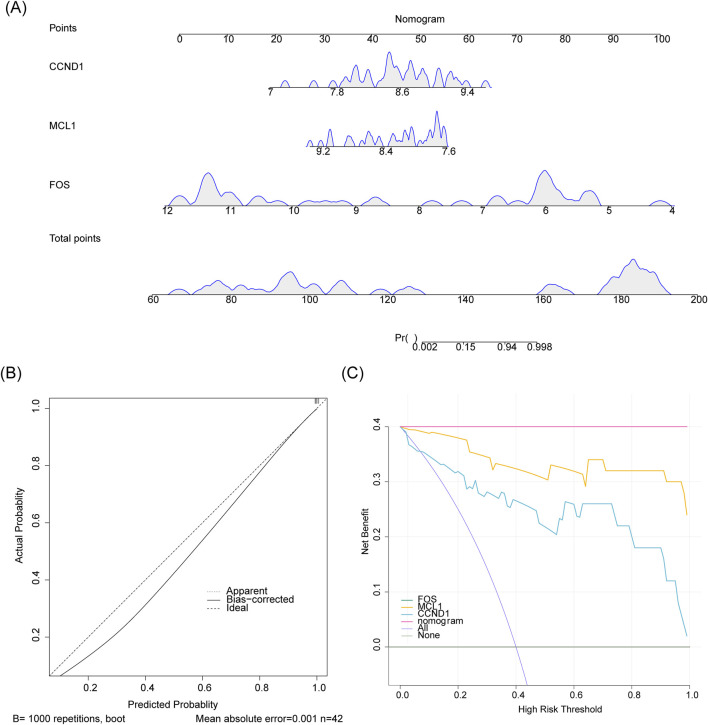
Evaluation of the nomogram predictive model. **(A)** A nomogram constructed using hub genes to estimate the risk of IgAN development. **(B)** Calibration plot assessing the agreement between predicted probabilities and observed outcomes. **(C)** Decision curve analysis (DCA) evaluating the clinical net benefit of the model.

A nomogram incorporating FOS, MCL1, and CCND1 was developed to evaluate the predictive capacity of these genes for IgAN (Figure 5A). The calibration curve showed close alignment between model-predicted outcomes and the ideal calibration curve, with both closely approximating the reference line of perfect prediction (slope = 1), indicating high predictive accuracy (Figure 5B). DCA revealed that the nomogram provided greater net benefit across a range of threshold probabilities compared to alternative strategies, highlighting its clinical utility (Figure 5C).

### 3.6 Enrichment pathway of potential key genes

**FIGURE 6:**
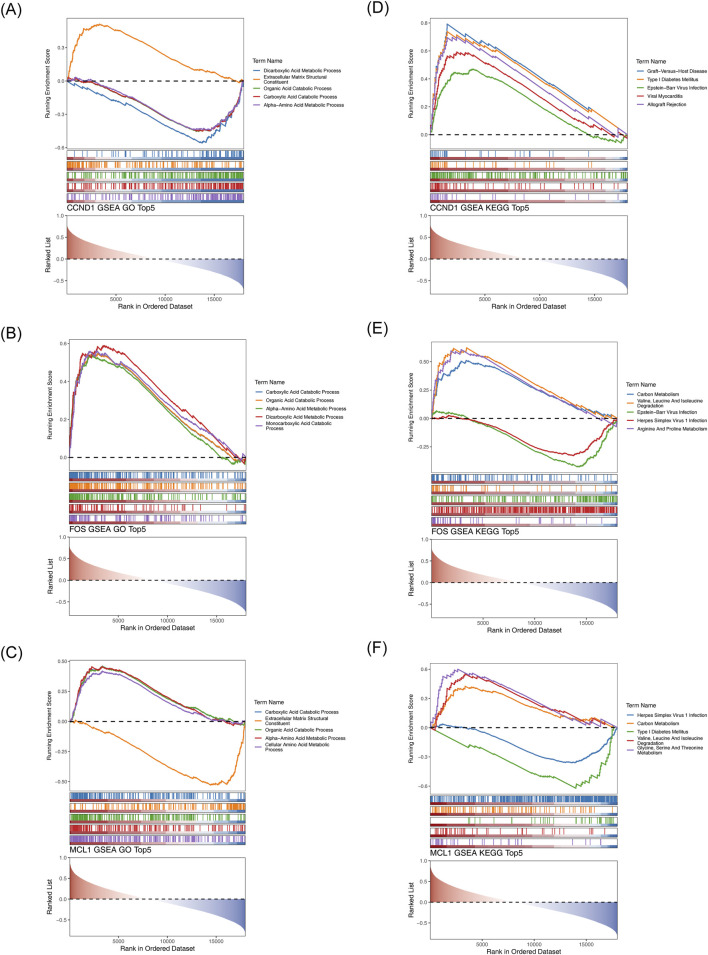
Gene Set Enrichment Analysis (GSEA) of hub genes. **(A,B)** Significantly enriched Gene Ontology terms and KEGG pathways associated with CCND1. **(C,D)** GO biological themes and KEGG signaling routes linked to FOS. **(E)** Enriched GO categories for MCL1. **(F)** KEGG pathway terms showing significant enrichment in relation to MCL1.

GSEA revealed a significant association between CCND1 and 1,020 GO terms, particularly those related to the catabolism of organic acids, metabolism of dicarboxylic acids, and metabolic processes involving α-amino acids (Figure 6A, Online Resource 3). CCND1 also showed substantial enrichment in 175 KEGG pathways, encompassing biological processes such as allograft rejection, Epstein-Barr virus infection, and mechanisms involving cell adhesion molecules (Figure 6B, Online Resource 4). GSEA indicated that FOS was prominently associated with 893 GO pathways, including the catabolic processes of organic acids, metabolism of dicarboxylic acids, and breakdown of carboxylic acids (Figure 6C, Online Resource 5). Additionally, FOS was significantly enriched in 162 KEGG pathways, including Epstein-Barr virus infection, arginine and proline metabolism, and carbon metabolic pathways (Figure 6D, Online Resource 6). Enrichment analysis showed that MCL1 participated in 651 GO pathways, especially those involving carboxylic acid catabolism, organic acid breakdown, and cellular amino acid metabolic processes (Figure 6E, Online Resource 7). MCL1 was also implicated in 129 KEGG pathways, including carbon metabolism and the metabolism of glycine, serine, and threonine (Figure 6F, Online Resource 8). Collectively, these results suggest that all potential key genes are functionally linked to dicarboxylic acid and cellular amino acid metabolic pathways.

### 3.7 Immune cell analysis for IgAN

**FIGURE 7:**
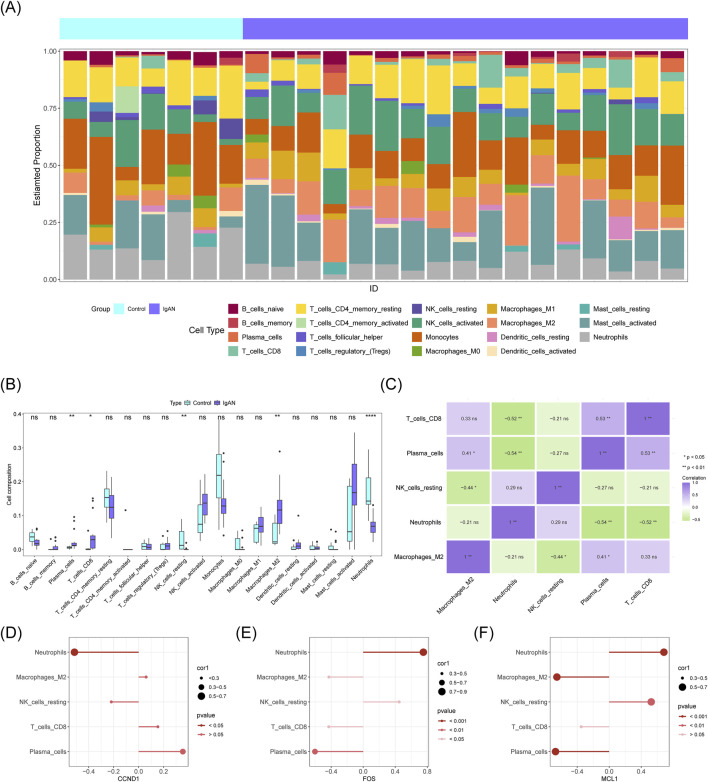
Analysis of immune cell infiltration. **(A)** Proportional abundance of 19 distinct immune cell populations. **(B)** Differentially abundant immune cell types comparing IgAN samples with control groups. **(C)** Correlation matrix illustrating interrelationships among various immune cells. **(D–F)** Associations between hub gene expression levels and immune cell infiltration.

Immune infiltration profiling was performed to compare the abundance of 19 immune cell types between IgAN and control groups, excluding 3 cell types with zero infiltration levels (Figure 7A). Significant differences were observed in five immune cell populations: plasma cells, CD8^+^ T cells, resting natural killer cells (Tang et al., 2021), M2 macrophages, and neutrophils (Figure 7B). A positive correlation was found between plasma cells and CD8^+^ T cells (cor = 0.53, *P* < 0.01), while the strongest negative correlation occurred between neutrophils and plasma cells (cor = −0.54, *P* < 0.01) (Figure 7C). Additionally, CCND1 showed a significant negative correlation with neutrophils (cor = −0.51, *P* < 0.05) (Figure 7D, Online Resource 9). FOS exhibited a significant negative correlation with plasma cells (cor = −0.60, *P* < 0.01) and a strong positive correlation with neutrophils (cor = 0.75, *P* < 0.01) (Figure 7E, Online Resource 9). Likewise, MCL1 demonstrated a marked negative association with plasma cells (cor = −0.67, *P* < 0.01) and a pronounced positive correlation with neutrophils (cor = 0.68, *P* < 0.01) (Figure 7F, Online Resource 9). These results suggest that the expression of potential key genes may be influenced by the abundance of plasma cells and neutrophils.

### 3.8 Molecular regulatory network analysis

**FIGURE 8:**
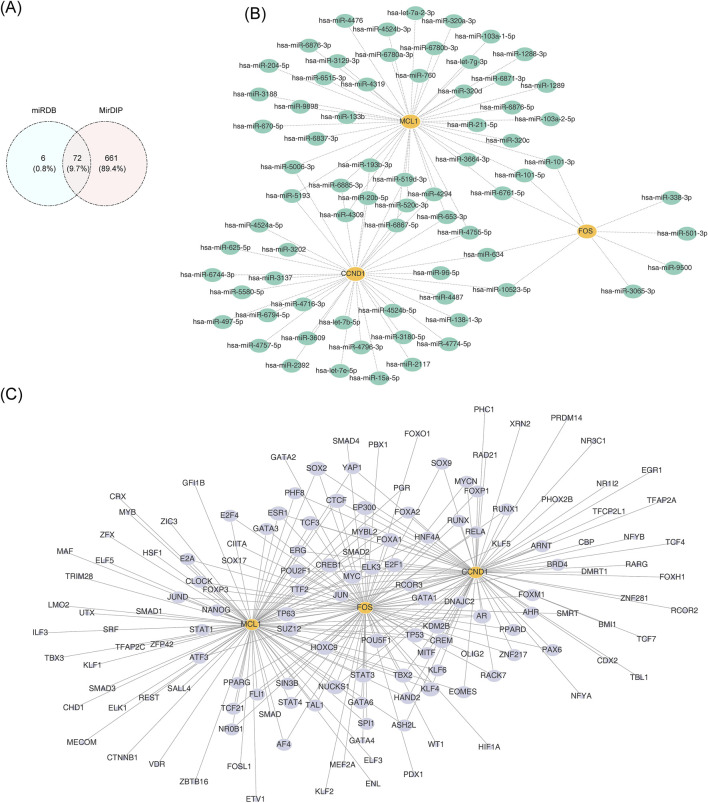
Molecular regulatory circuitry. **(A)** Venn diagram depicting miRNAs predicted to target the identified hub genes. **(B)** Network of miRNA–mRNA interactions, with green nodes indicating key regulatory miRNAs and yellow nodes denoting hub genes. **(C)** Transcriptional regulatory network of transcription factors (TFs) and target mRNAs. Yellow nodes represent hub genes; gray nodes correspond to TFs.

A total of 72 miRNAs were identified through the intersection of predictions from two independent databases. Among them, 37 miRNAs were predicted to target CCND1, 9 to target FOS, and 43 to target MCL1 (Figure 8A). Intersection analysis revealed that MCL1 and CCND1 shared 12 common miRNA regulators, including hsa-miR-6885-3p; MCL1 and FOS co-targeted three miRNAs, such as hsa-miR-101-5p; and FOS and CCND1 jointly targeted two miRNAs: hsa-miR-634 and hsa-miR-10523-5p (Figure 8B). Additionally, the TF regulatory network analysis indicated that 76 TFs potentially regulate CCND1, 75 target FOS, and 89 are associated with MCL1 (Figure 8C).

### 3.9 Analysis of potential key gene expression in key cells

**FIGURE 9:**
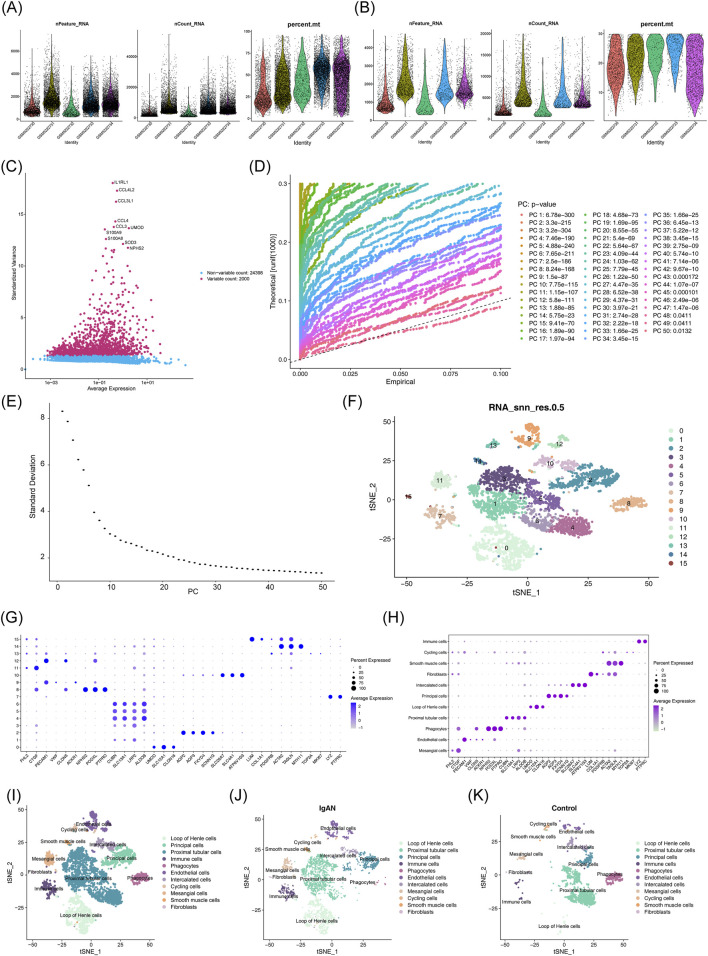
Processing and analysis of single-cell RNA sequencing data. **(A)** Violin plots displaying feature counts, UMI counts, and mitochondrial gene percentages pre- and post-quality filtering. **(B)** Distribution of gene expression levels across cells. **(C)** Top ten genes exhibiting the highest variability in expression. **(D,E)** Principal component analysis (PCA) and associated elbow plot utilized for dimensionality reduction. **(F)** t-distributed stochastic neighbor embedding (t-SNE) visualization identifying 16 distinct cell clusters. **(G)** Bubble chart illustrating marker gene expression patterns across different cellular clusters. **(H)** Annotated bubble plot representing cell cluster identities. **(I)** t-SNE projection with cluster annotation labels. **(J)** Cell type composition within the IgAN patient group. **(K)** Annotation of cell types present in the control cohort.

Following quality control of the cRNA-seq data, 5,272 cells and 26,398 genes were retained for downstream analysis (Figures 9A,B). The top 2,000 most variable genes were selected, with the ten most variable, including IL1RL1, CCL4L2, and CCL3L1, highlighted (Figure 9C). Based on the JackStraw plot and elbow plot, PCs beyond the first 30 showed stabilized variance, and thus, the first 30 PCs were selected for further analysis (Figures 9D,E). A total of 16 distinct cell clusters were identified (Figure 9F; Supplementary Table S1). Cluster annotation was performed using a bubble plot to visualize marker gene expression patterns across clusters and corresponding cell types (Figures 9G–I). The resulting 11 annotated cell populations were visualized according to their sample group origins (Figures 9J,K).

**FIGURE 10:**
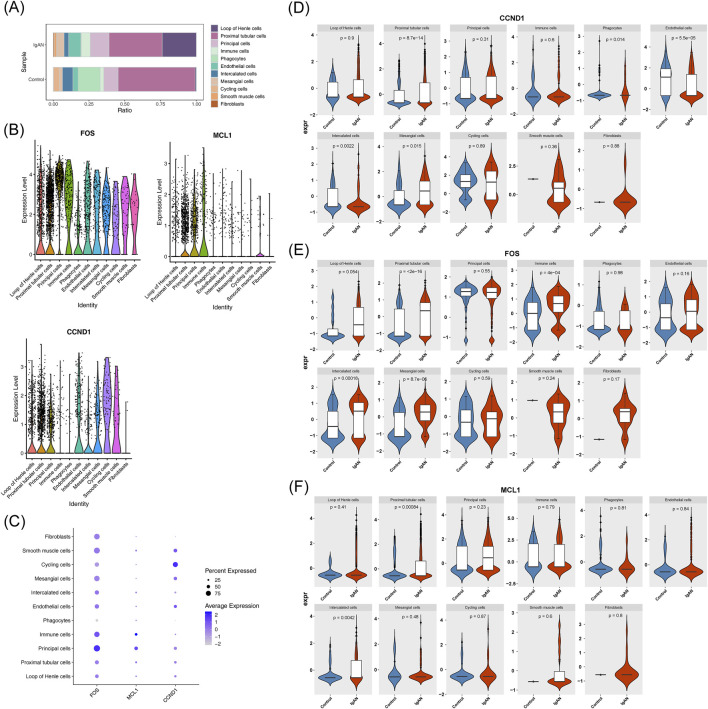
Identification of pivotal cell populations. **(A)** Proportional distribution of annotated cell types in IgA nephropathy and control samples. **(B,C)** Expression patterns of hub genes across distinct cellular subtypes. **(D–F)** Differential expression profiles of potential key genes specifically in proximal tubular cells and intercalated cells.

Analysis of cellular composition revealed that proximal tubular cells and loops of Henle cells were the predominant populations in the IgAN group (Figure 10A). Expression profiling showed elevated levels of FOS and MCL1 in principal cells, while CCND1 expression was highest in cycling cells (Figures 10B,C). Significant differences in the expression of all potential key genes were observed between IgAN and control samples, particularly within proximal tubular cells and intercalated cells (Figures 10D–F). As a result, these 2 cell types were selected for subsequent pseudotime trajectory analysis.

**FIGURE 11:**
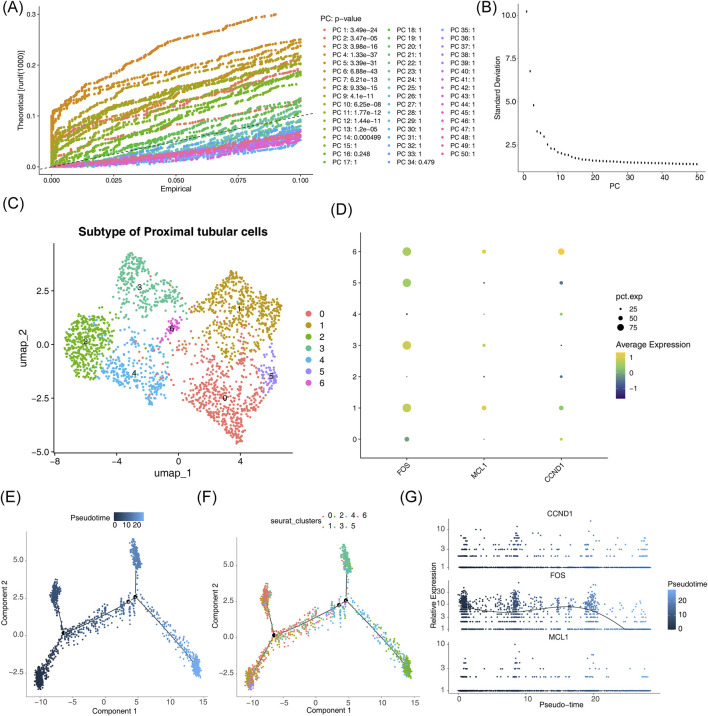
Pseudotemporal trajectory analysis of proximal tubular cells. **(A–C)** Subgroup clustering and corresponding t-SNE visualizations. **(D)** Expression levels of hub genes identified across distinct subgroups. **(E)** Reconstructed developmental trajectory based on pseudotime inference. **(F)** Distribution of cellular subgroups along the inferred pseudotemporal axis. **(G)** Dynamic changes in potential key gene expression throughout the differentiation process.

Proximal tubular cells were categorized into seven distinct subclusters (Figures 11A–C), with subclusters 1 and 6 exhibiting the highest expression levels of all potential key genes analyzed (Figure 11D). Pseudotime trajectory analysis revealed a differentiation continuum within proximal tubular cells, represented by a gradient from dark to light blue (Figure 11E). Subclusters 2 and 3 were predominantly present in the terminal phase of cellular differentiation, while subclusters 0 and 1 were more abundant in the early stages (Figure 11F). Throughout the developmental progression of proximal tubular cells, FOS expression exhibited dynamic fluctuations but remained elevated overall, followed by a sharp decline in the later stages. In contrast, expression patterns of CCND1 and MCL1 remained relatively stable across the differentiation timeline (Figure 11G).

**FIGURE 12:**
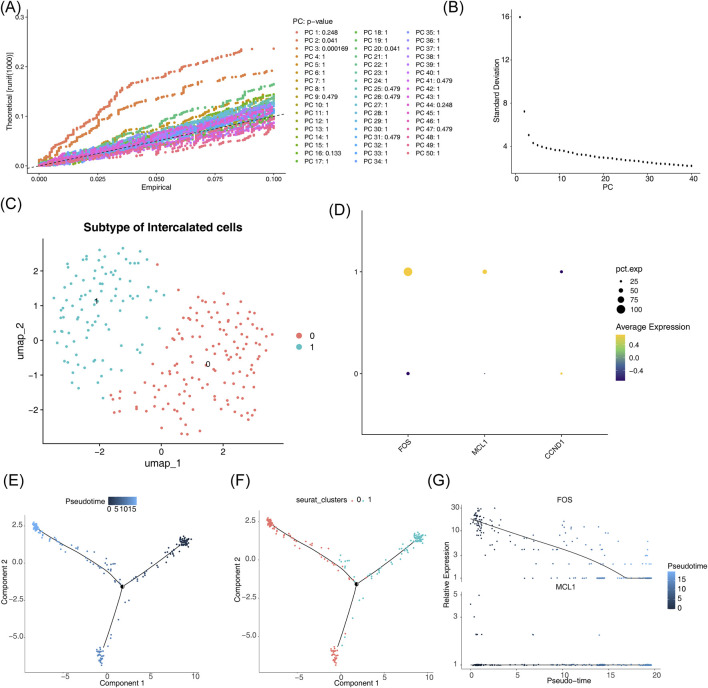
Pseudotime trajectory reconstruction for intercalated cells. **(A–C)** Identification of cellular subgroups and their representation in t-SNE space. **(D)** Variations in hub gene expression among different subgroups. **(E)** Inferred developmental progression using pseudotime analysis. **(F)** Placement of cellular subtypes across the pseudotemporal continuum. **(G)** Temporal alterations in critical gene expression during cellular differentiation.

Similarly, intercalated cells were partitioned into two distinct subpopulations (Figures 12A–C). Subgroup 1 exhibited the highest expression levels for all potential key genes examined (Figure 12D). Pseudotime analysis revealed a differentiation trajectory within intercalated cells, represented by a color gradient transitioning from dark to light blue (Figure 12E). Subgroup 0 was more prominent during early differentiation, whereas subgroup 1 became more abundant in later stages (Figure 12F). Throughout intercalated cell development, FOS expression showed a consistent downward trend, while MCL1 levels remained relatively stable (Figure 12G).

### 3.10 Binding energy analysis of potential key genes and drugs

**FIGURE 13:**
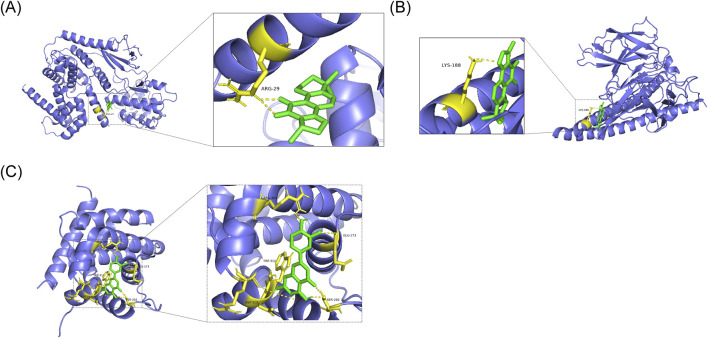
Molecular docking analysis between hub genes and bioactive compounds. **(A)** Predicted binding mode of cryptotanshinone with CCND1. **(B)** Interaction model of tanshinone IIA docked to FOS. **(C)** Binding conformation of luteolin complexed with MCL1. Hydrogen bonding interactions are indicated.

Based on OB screening of active compounds, cryptotanshinone, tanshinone IIA, and luteolin were selected for molecular docking studies (Table 3). In the CCND1 protein structure, the cryptotanshinone molecule formed a hydrogen bond with the ARG-29 residue, resulting in a binding energy of −7.7 kcal/mol (Figure 13A; Supplementary Tables S2,S3). In the FOS protein structure, the tanshinone IIA molecule established a hydrogen bond interaction with the LYS-188 residue, yielding a binding energy of −7.5 kcal/mol (Figure 13B; Supplementary Tables S4,S5). For MCL1, multiple residues—including ARG-303, ARG-310, ARG-313, TRP-312, SER-202, and GLU-173—participated in hydrogen bonding with luteolin, producing a binding energy of −6.9 kcal/mol (Figure 13C; Supplementary Tables S6,S7). Further molecular docking demonstrated that the binding energy between tanshinone IIA and FOS was superior to that of the negative control, N-nitrosodiethylamine, with FOS (−4.0 kcal/mol) (Supplementary Tables S8). This finding suggested a specific interaction between tanshinone IIA and FOS. These molecular docking results suggest strong binding interactions between the potential key genes and active compounds. It is important to note that the current docking results represent only computer-simulated predictions of binding affinity and do not constitute direct evidence of biological function. Therefore, the efficacy of drug function must be validated experimentally.

**TABLE 3:** Results of oral bioavailability (OB) screening for active compounds

Potential key genes	TCMSP compound ID	Active ingredient	Oral bioavailability (OB, %)
CCND1	MOL007088	Cryptotanshinone	52.34196
FOS	MOL007154	Tanshinone IIA	49.8873
MCL1	MOL000006	Luteolin	36.16263

### 3.11 RT-qPCR result

**FIGURE 14:**
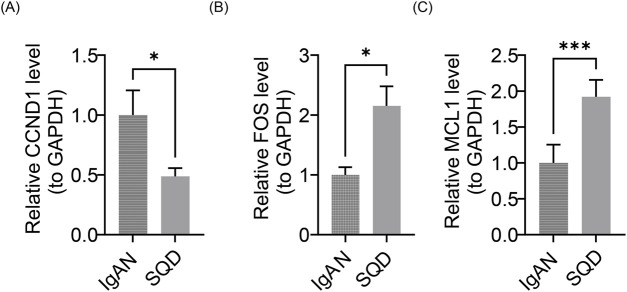
Quantitative real-time polymerase chain reaction (RT-qPCR) analysis confirming the expression of pivotal genes. **(A)** Comparison of FOS transcript abundance between IgA nephropathy (IgAN) specimens and those administered SQD. **(B)** MCL1 mRNA expression levels in IgAN and following SQD intervention. **(C)** Expression profiles of CCND1 in the context of IgAN and after treatment with SQD.

*In vitro* experiments showed that expression levels of FOS (*P* < 0.05) (Figure 14A) and MCL1 (*P* < 0.001) (Figure 14B) were significantly reduced in the IgAN group compared to the SQD-treated group. Conversely, CCND1 expression was markedly lower in the SQD-treated group relative to the IgAN group (*P* < 0.05) (Figure 14C). Together with bioinformatic findings, these results highlight the substantial protective effect of SQD against IgAN.

## 4 Discussion

IgAN is a prevalent primary glomerular disorder, but its underlying pathogenic mechanisms remain incompletely understood. Renal biopsy is currently the gold standard for diagnosis. In a subset of patients, IgAN follows an aggressive clinical course, potentially leading to rapid renal failure and poor prognostic outcomes.

This study integrates bioinformatics and network pharmacology approaches to propose potential mechanisms of SQD in treating IgAN. The analysis results indicate that three potential key genes—FOS, MCL1, and CCND1—may serve as core regulatory factors in this process. A nomogram prediction model was constructed to evaluate the diagnostic potential of these genes in IgAN.

FOS (Fos proto-oncogene) is an immediate-early response gene that regulates cell proliferation, differentiation, and transformation. The leucine zipper motif encoded by FOS facilitates dimerization with JUN family proteins, forming the activator protein 1 (AP-1) transcription factor complex, which plays a critical role in cellular processes such as proliferation, differentiation, and oncogenic transformation (Song et al., 2023). Under certain conditions, FOS expression has been linked to the induction of apoptotic cell death (Zhou et al., 2022; Ren et al., 2024; Noor et al., 2021).

Previous studies (Zhou et al., 2022) suggest that FOS expression levels could serve as a discriminative marker between patients with IgAN and healthy controls. Another study (Al Mehedi Hasan et al., 2022) employed bioinformatic models integrating machine learning and statistical methods, identifying FOS as a potential key gene in IgAN. The study revealed that FOS expression exhibited dynamic variations during proximal tubular cell development, remaining elevated overall before sharply declining in later differentiation stages. In intercalated cells, FOS expression decreased progressively throughout development. These findings suggest that FOS may serve as a valuable biomarker and key participant in the pathophysiology of IgAN, and its clinical application value is worthy of further exploration.

MCL1 (Myeloid Cell Leukemia Sequence 1) encodes an anti-apoptotic protein that interacts with BCL-2 family members—including pro-apoptotic proteins such as BAX and BAK—to regulate programmed cell death. It is widely expressed across normal tissues and plays a critical role in the maturation and differentiation of T cells, B lymphocytes, and macrophages. Targeted inhibitors of MCL1 have shown promising efficacy in preclinical animal studies (Li et al., 2021; Ma et al., 2023; Patel et al., 2022).

Given the significant genetic heterogeneity observed in cancers, therapeutic responses in malignant cells can vary considerably. MCL1 is emerging as a potential biomarker and therapeutic target in renal cell carcinoma, with the ability to provide survival benefits that may promote the retention of effective treatment strategies (Li et al., 2021).

The therapeutic mechanism of Schisandra Chinensis Mixture (SM) in diabetic nephropathy was explored through integrated network pharmacology and bioinformatics analysis. Potential molecular targets and key signaling pathways of SM were validated *in vivo*, with MCL1 identified as a central regulatory gene (Ma et al., 2023). This study is the first to identify MCL1 as a potential key gene associated with IgAN. Although there is currently no direct research confirming the relationship between MCL1 and IgAN, given its role in immune cell regulation and kidney diseases, MCL1 may play a significant role in the immune response and renal damage in IgAN. Further exploration of its potential as a biomarker or therapeutic target is warranted.

Large-scale genetic analyses (Patel et al., 2022) have shown that the deletion of the VHL gene occurs in approximately 90% of clear cell renal cell carcinomas (ccRCCs), as demonstrated by experimental models, functional genomic studies, and patient-derived samples. The study further highlighted that enhancer-driven activation of cyclin D1 (CCND1) gene expression plays a pivotal role in oncogenic signaling pathways.

CCND1 (cyclin D1) (Jiang et al., 2020) is a proto-oncogene located at the 11q13 locus of chromosome 11. In DN, CCND1 contributes to disease progression by modulating cellular proliferation and apoptotic processes. Its expression is regulated by miR-141-3p and SNHG16. CCND1 associates with CDK4 or CDK6 to form an active holoenzyme, which phosphorylates tumor suppressor proteins, facilitating the release of E2F TF. This activity drives the transition of cells from the G1 to the S phase, thus regulating progression through the cell cycle (Jiang et al., 2020; Lei et al., 2022; Guo et al., 2023).

In IgAN, CCND1 plays a role in mesangial cell proliferation and fibrotic processes through modulation of miR-93/106b, indicating its potential involvement in IgAN pathogenesis (Ma et al., 2021). Based on these findings, it was hypothesized that CCND1 could influence the progression of IgAN by regulating cellular proliferation and apoptotic pathways.

Discussion on the immune microenvironment and potential key gene correlations: Immune infiltration analysis revealed five immune cell types with differential abundance in IgAN: plasma cells, CD8^+^ T cells, resting NK cells, M2 macrophages, and neutrophils. Correlation analysis between differentially abundant immune cells and key genetic factors indicated that the three potential key genes exhibited stronger correlations with plasma cells and neutrophils (Mathur et al., 2023; Maixnerova et al., 2022; Kronbichler et al., 2023).

The pathogenesis of IgAN is characterized by glomerular deposition of immune complexes formed by galactose-deficient immunoglobulin A1 (Gd-IgA1) and its corresponding autoantibodies. This deposition triggers complement-mediated inflammation within the glomeruli, potentially leading to end-stage kidney disease (ESKD). Interventions targeting B cells and CD38-positive plasma cells—either by inhibiting their activation or inducing depletion—can attenuate complement-driven inflammation and the generation of pathogenic antibodies, offering potential therapeutic strategies for IgAN (Maixnerova et al., 2022).

Previous studies investigating the OB of active compounds and their binding affinities to potential key genes have validated cryptotanshinone as a bioactive constituent with therapeutic effects on renal injury (Yang W. et al., 2022). Cryptotanshinone has shown particular promise for therapeutic use, especially in the complex pathophysiology of postmenopausal osteoporosis with concurrent renal impairment.

In addition, tanshinone IIA has been identified as a critical modulator in oxidative stress-triggered pyroptosis (Wu Q. et al., 2023). In DN—a condition associated with significant health risks—sustained hyperglycemia exacerbates oxidative stress levels in renal compartments, triggering pyroptosis and contributing to progressive kidney injury. Tanshinone IIA was found to suppress pyroptosis induced by oxidative stress, reduce renal cell mortality, and preserve the structural and functional integrity of kidney tissues.

Luteolin (Zhang et al., 2024b) primarily protects genetic material within renal cells by mitigating oxidative DNA damage induced by cadmium. Additionally, it reverses cadmium-induced disruption of autophagic flux, facilitating the restoration of normal autophagy. Through this dual mechanism, luteolin enhances cellular defense and mitigates the detrimental effects of cadmium on renal cells.

Through single-cell analysis, this study identified 2 cell types—proximal tubule cells and intercalated cells—as potentially associated with IgAN, given their association with FOS (elevated FOS expression levels were observed in these cell populations). Notably, due to the limited sample size (4 IgAN patients and 1 healthy control), the identified cell populations are only preliminary findings and require further validation in larger cohorts.

Research suggests that proximal tubular cells undergo significant changes during the early stages of the disease, and potential intercellular communication pathways may exist between the glomerular and tubular compartments. The functional importance of key signaling pathways, such as the Slit-Robo pathway, in cellular crosstalk has been confirmed through *in vitro* cell culture systems. Therefore, our findings offer novel molecular insights critical for understanding the onset of glomerular injury in IgAN (Zambrano et al., 2022; Liu et al., 2023b).

By employing bioinformatics and network pharmacology, three potential key genes and two essential cell types involved in the therapeutic mechanism of SQD in IgAN were identified. This investigation sheds light on SQD’s mode of action, providing new perspectives on its potential application in IgAN treatment and supporting the development of targeted therapeutic strategies and drug development.

However, this study has several limitations, notably the lack of validation through *in vitro* experiments. Future work will focus on these potential key genes and cellular subtypes, utilizing both *in vivo* and *in vitro* mechanistic studies to further elucidate their roles, ultimately establishing a more robust theoretical foundation for IgAN therapy. Furthermore, due to the limited sample size of the RT-qPCR experiments, the findings of this study should be considered preliminary and require further validation in a larger-scale sample cohort.

## 5 Conclusion

This study demonstrates that three genes—FOS, MCL1, and CCND1—may be involved in the mechanism by which SQD modulates immunoglobulin A nephropathy (IgAN), and they may be associated with diagnostic relevance and metabolic pathway involvement. *In vitro* experiments indicate that SQD may exert a cytoprotective effect, providing a promising strategy for the treatment of IgAN.

## Data Availability

The data that support the findings of this study are openly available in the Gene Expression Omnibus (GEO) database [GSE93798 and GSE37460) [http://www.ncbi.nlm.nih.gov/geo/].

## Supplementary material

SUPPLEMENTARY TABLE S8 Detailed information on the molecular docking between N-nitrosodiethylamine and FOS.
